# Transcultural Adaptation of and Theoretical Validation Models for the Spanish Version of the Nurses’ Global Assessment of Suicide Risk Scale: Protocol for a Multicenter Cross-sectional Study

**DOI:** 10.2196/39482

**Published:** 2022-09-21

**Authors:** Maria Elena Garrote-Cámara, Raúl Juárez-Vela, Teresa Sufrate-Sorzano, Angela Durante, Paolo Ferrara, Stefano Terzoni, Jesús Pérez, Iván Santolalla-Arnedo

**Affiliations:** 1 Department of Nursing, Research Group in Care University of La Rioja Logroño Spain; 2 Group of Research in Sustainability of the Health System, Biomedical Research Center of La Rioja Rioja Health Service Government of La Rioja Logroño Spain; 3 San Paolo Bachelor School of Nursing University of Milan Milano Italy; 4 Cambridgeshire and Peterborough NHS Foundation Trust Cambridge Cambridge United Kingdom

**Keywords:** mental health, suicide, psychiatric nursing, Spanish, translate, translation, scale, measurement, assessment, adapt, adaptation, cultural, transcultural, suicidal, nurse, nursing, psychiatric, public health, prevention, treatment, risk, development, lethal, patient, scientific literature, variables, reliability, validate, validity, tool, Nurse´s Global Assessment of Suicide Risk, psychometric

## Abstract

**Background:**

The use of validated instruments means providing health professionals with reliable and valid tools. The Nurses’ Global Assessment of Suicide Risk (NGASR) scale has proven to be valid and reliable in supporting the nursing evaluation of suicide risk in different languages and cultural environments.

**Objective:**

The aims of our study are to translate and adapt the NGASR scale for the Spanish population and evaluate its psychometric properties in patients with suicide risk factors.

**Methods:**

The translation, adaptation, and modeling of the tool will be performed. The sample will include 165 participants. The psychometric analysis will include reliability and validity tests of the tool’s internal structure. The tool’s reliability will be assessed by exploring internal consistency and calculating the Cronbach α coefficient; significance values of .70 or higher will be accepted as indicators of good internal consistency. The underlying factor structure of the Spanish version of the NGASR scale will be assessed by performing an exploratory factor analysis. The Kaiser-Meyer-Olkin measure of sample adequacy and the Bartlett sphericity statistic will be calculated beforehand. For the latter, if *P* is <.05 for the null hypothesis of sphericity, the null hypothesis will be rejected.

**Results:**

Participants will be recruited between April 2022 and December 2022. Our study is expected to conclude in the first quarter of 2023.

**Conclusions:**

We hope to find the same firmness that colleagues have found in other countries in order to consolidate and promote the use of the NGASR tool in the Spanish population. The prevention and treatment of suicidal behavior require holistic, multidisciplinary, and comprehensive management.

**International Registered Report Identifier (IRRID):**

PRR1-10.2196/39482

## Introduction

Nowadays, suicide is a public health issue for which prevention and treatment must be prioritized by macromanagement, mesomanagement, and micromanagement in health programs worldwide [1]. Suicidal behavior is determined by the complex interplay among factors that pose a risk for the development of lethal behavior, risk factors and predisposing circumstances that may determine and precipitate suicidal behavior, and protective factors that provide life-sustaining safety [2,3].

The World Health Organization estimates that almost 800,000 people commit suicide every year worldwide, and for each of these suicides, it is estimated that there are 20 suicide attempts [4]. Therefore, we can estimate that there are more than 16 million suicide attempts every year worldwide. Suicide attempts are repeated by 15% to 30% of patients within 1 year, and almost 2% end up committing suicide within 5 to 10 years of their initial suicide attempt [5]; suicide attempts are therefore the most relevant risk factor [6]. Internationally, the countries with the highest suicide rates are Lithuania, South Korea, and Slovenia, where the suicide rate exceeds 30 cases per 100,000 inhabitants. Greece, Turkey, and South Africa appear at the bottom of the list, with suicide rates of less than 4 deaths per 100,000 inhabitants [7]. In Spain, more than 3500 people commit suicide every year, and this has been on an upward trend since 2014, with the suicide rate exceeding 10 suicides per 100,000 inhabitants [8]. The highest suicide rates per inhabitant and per autonomous community are in Asturias, Galicia, and Murcia. Cantabria, Ceuta, and Melilla have the lowest rates. Both nationally and internationally, hanging and jumping from a height are the most commonly selected methods [7].

Risk assessment scales for suicidal behavior are instruments that are available to health care providers in clinical practice and research. These instruments guarantee that quality standards are met in the results of their measurements and allow for the systematizing and universalizing of perceived observations. In order to support health care professionals in systematizing and assessing suicidal risk, it is important to determine the most appropriate intervention, as well as how to record cases and the care provided, and use validated suicide risk assessment scales that always require prior consultations with the patients and clinical interviews [8,9]. Among the most commonly used scales are the Horowitz Suicide Risk Questionnaire [10], Beck Hopelessness Scale [11], Beck Scale for Suicide Ideation [12], Hamilton Depression Inventory [13], Plutchik Suicide Risk Scale [14], Reasons for Living Inventory [15], and the Nurses' Global Assessment of Suicide Risk (NGASR) scale [8]. In addition, after the validation of such scales for specific populations, they can be converted into a web-based format and further developed for use on a web-based platform that facilitates registration and evaluation for health care professionals [16,17].

The NGSAR scale, which is noted for its ease of use [18], has been included as a suitable tool for assessing suicide risk in the Registered Nurses Association of Ontario’s best practice manuals [19]. In Spain, nurses are the first line of care; therefore, having a scale with good psychometric properties in assessing suicide risk has become essential [20]. Taking into account the relevance and wide use of the NGASR in clinical practice and research and the fact that the scale has been validated in different languages (eg, German, Mandarin Chinese, Portuguese, Korean, and Italian) with good validity and reliability [8,18,21-29], the aims of this work are to translate and adapt this scale in Spanish—the second most spoken language in the world [30]—and evaluate its psychometric properties in patients with risk factors and suicidal behaviors.

## Methods

### Search Strategy

**Figure 1:**
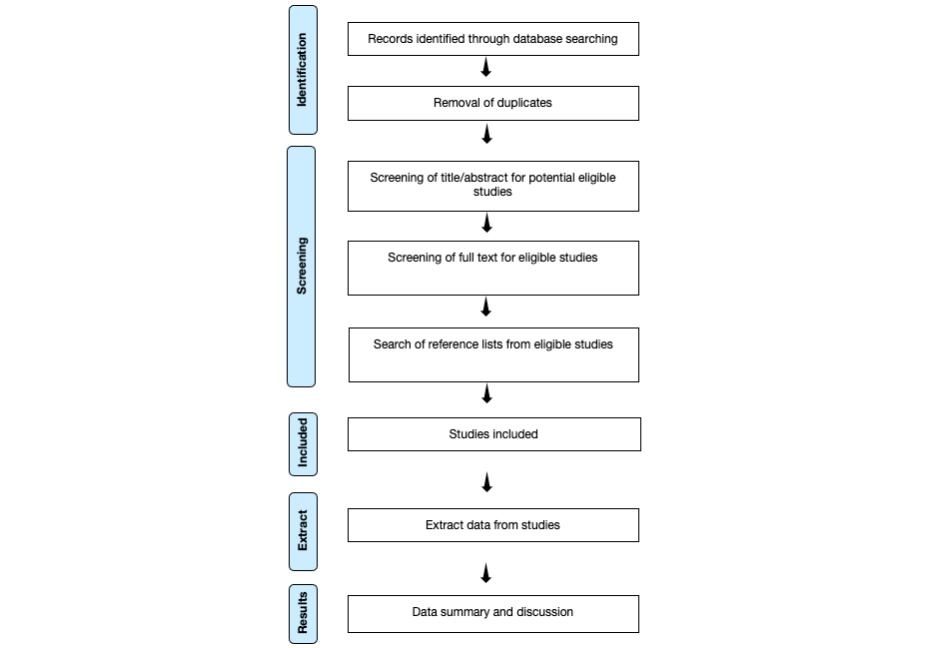
Flowchart of the protocol.

Initially, a review of the literature will be conducted in order to learn about previous adaptions in different languages and cultural environments and about the psychometric characteristics of the NGASR. A search for articles indexed in major health science databases will be performed. In addition, the bibliographic references of the included reviews will be searched. The results will be assessed for inclusion by 2 independent reviewers, and an assessment of methodological quality and data extraction will be performed. The search for scientific literature will be conducted based on the following keywords: *Suicide Attempted*, *Nurse*, *Risk Assessment*, *Risk*, *Scale*, and *NGASR*. They will be combined by means of Boolean operators (“AND” and “OR”) and adapted to each database in a specific way. The literature review procedure that will be followed in our study is described in Figure 1.

### Translation, Adaptation, and Modeling

Before testing its psychometric properties, the NGASR scale will be translated and culturally adapted from its original English version to Spanish. The NGASR scale was developed by Cutcliffe and Barker [8] in 2004 and consists of 15 items, of which each has a score of 1 or 3 points. Variables such as hopelessness, depressive symptoms, suicidal plans, the grief process, and a history of previous suicide attempts are scored with 3 points, while the rest of the variables are scored with 1 point. A final score of 0 to 5 points indicates low suicide risk, scores of 6 to 8 indicate intermediate suicide risk, scores of 9 to 11 indicate high suicide risk, and scores of ≥12 indicate a very high level of suicide risk. These items were designed so that during interviews, nursing staff can collect the necessary information for each of the variables (the scale is heteroadministered).

We will follow the guidelines published by Beaton et al [31], which divide the process into the following six steps: translation, synthesis, back-translation, back-translation synthesis, an expert review of the translated version, and pretesting.

For this purpose, a nursing professional expert in mental health and a bilingual nursing professional will each lead 1 of 2 independent groups that will carry out the translation and adaptation process for an initial Spanish version. As such, 2 initial Spanish versions will be developed (*Version 1–group A* and *Version 1–group B*). After comparing the two versions and in order to reach consensus on the discrepancies, the criteria of both groups will be unified, and a final version of the document will be created in Spanish (ie, the NGASR–Spanish version [NGASR-SPN]), which will be back-translated into English by an official entity that will certify this process. The back-translated version (the NGASR-SPN) will be provided to the original authors to confirm the accuracy of the instrument.

The NGASR-SPN will be evaluated by 10 mental health specialist nurses with more than 5 years of experience.

### Participants in the Validation Process

A sample of 165 patients (10 patients for each item on the NGASR plus 10% to avoid possible losses) who are admitted to one of the units within La Rioja’s mental health network. These units will comprise primary care mental health units, day hospitals, partial hospitalization units, short stay hospitalization units, medium stay hospitalization units, and long stay hospitalization units.

The inclusion criteria for participation in the study will be (1) people aged over 18 years; (2) patients diagnosed with a mental disorder according to the clinical descriptions and diagnostic guidelines of the *International Classification of Diseases, 11th Revision* [32] and the *Diagnostic and Statistical Manual of Mental Disorders, Fifth Edition* [33]; and (3) patients undergoing follow-up or treatment in one of the mental health departments of La Rioja Health Service.

The exclusion criteria will be (1) civilly incapacitated patients, (2) patients with cognitive or perceptual impairments, and (3) patients whose first language is not Spanish.

The sample size—165 patients—was estimated according to the criteria for a factor analysis with a minimum of 10 patients for each item on the NGASR [34], and another 10% will be recruited to avoid possible losses.

### Data Analysis

The psychometric analyses of the Spanish version of the NGASR will include tests of the reliability and validity of its internal structure. The reliability of the scale will be assessed by exploring internal consistency and calculating the Cronbach α coefficient; significance values of .70 or higher will be accepted as indicators of good internal consistency [35].

The underlying factor structure of the NGASR-SPN scale will be assessed by performing an exploratory factor analysis. To assess the relevance of performing an exploratory factor analysis on the sample, the Kaiser-Meyer-Olkin measure of sample adequacy and the Bartlett sphericity statistic will be calculated beforehand. The adequacy of the sample for these analyses will be determined with optimal values for Kaiser-Meyer-Olkin measure, and in the case of the Barlett test of sphericity, if *P* is <.05 for the null hypothesis of sphericity, the null hypothesis will be rejected to ensure that the correlation matrix is adequate for obtaining a factor model that is able to properly describe the data.

The data will be coded and recorded in a computer format. Data processing and statistical calculations will be carried out with the SPSS Statistics software (IBM Corporation) [36].

### Ethics Approval

To carry out the validation of the NGASR-SPN scale for the Spanish population, prior authorization has been requested from the Ethics Committee for Research on Medicines in La Rioja (reference number: PI-467). The patients will be informed of the objectives and methodology of the work, and we will request them to provide their free, voluntary, and informed consent. We will guarantee data confidentiality and use the information obtained exclusively for research purposes in accordance with *Organic Law 3/2018, of December 5, on the Protection of Personal Data and Guarantee of Digital Rights* [37] and *Regulation (EU) 2016/679 of the European Parliament and of the Council of 27 April 2016 on the protection of natural persons with regard to the processing of personal data and on the free movement of such data* [38].

### Dissemination

The results obtained in the process of adapting the NGASR to the Spanish population will be made available to regional health services and university centers at the national level by the Biomedical Research Center of La Rioja, the University of La Rioja, and Salamanca Biomedical Research Institute. The results will also be disseminated in congresses of psychiatry and nursing that recognize and are interested in the health impacts of our study, as well as scientific journals with national and international impacts. We will follow the phases described in Table 1.

**Table 1:** Study phases.

Phases and activities		2021			2022	
		October	November	December	January	February
**Planning**						
	Contact the authors of the instrument	✓				
	Review the theoretical framework and search for bibliographical references	✓	✓			
	Analysis of the validation process developed in other countries		✓			
	Preparation of an informed consent document for patients	✓				
	Drafting of a document for the research ethics committee	✓				
	Elaboration of research protocol and acceptance by the authors	✓	✓			
	Research ethics committee authorization		✓			
**Implementation**						
	Briefing of experts on the translation process of the tool	✓				
	Comparison of the versions translated by both groups of experts and consensus		✓			
	Back-translation of the final version by a certified body		✓			
	Contact the authors to show them the translated version		✓			
	Briefing meeting with the nursing professionals who will evaluate the first translated version in practice		✓			
	Compilation of the study sample		✓	✓	✓	
	Statistical analysis				✓	
	Interpretation of the results obtained				✓	✓
	Organization of the data obtained				✓	✓
	Elaboration of the discussion and conclusion				✓	✓
	Presentation to the authors of the draft and review of contributions					✓
	Selection of the most appropriate journal for dissemination					✓
**Dissemination**						
	Submission of the work to a journal of scientific interest					✓
	Organization of a conference to present the results to the health network					✓
	Presentation of the validation process at a national or international congress of scientific interest					✓
	Working meeting on the process: aspects for improvement, strengths, weaknesses, and opportunities					✓

## Results

Participants will be recruited between April 2022 and December 2022. Our study is expected to conclude in the first quarter of 2023.

## Discussion

We believe that the results of our study can help prevent and manage suicidal behavior in the population, since the use of validated instruments means providing health professionals with reliable and valid tools. Several studies have demonstrated the robust properties of the NGASR scale in different languages and cultural environments. However, no study has validated the NGASR in Spanish—one of the most widely spoken languages in the world [30]. With our study, we hope to find the same firmness that colleagues have found in other countries in order to consolidate and promote the use of this assessment tool in the Spanish population. The scale must first be culturally adapted to the environment where it will be used, and then its psychometric characteristics must be remeasured [39].

Validating this scale in Spanish will provide a standardized suicide risk assessment instrument that can be used by nursing staff, be recorded in patients’ electronic medical records, and facilitate assistance and further research studies for preventive purposes. As the NGASR-SPN will be made available to health care providers in the first line of care, such as nurses, the scale will be a key tool in the work of any nurse in the Spanish population. Furthermore, a clear benefit of the validation of specific instruments in the field of health for a circumscribed context is the ability to compare the results obtained with those of studies that are carried out in other countries and use the same instrument. Such validation favors the universality of care, and the NGASR-SPN will result in less variability in nursing practices [40,41].

The magnitude of suicide is a serious public health problem; therefore, it is necessary to develop validated tools for its evaluation, with the ultimate goal of reducing the prevalence of suicidal behaviors.

The translation of the NGASR scale into Spanish will allow nurses to perform a more accurate assessment of suicide risk in Spanish-speaking countries, thus contributing to the provision of interventions aiming to prevent suicidal behaviors.

## Abbreviations

NGASR: Nurses’ Global Assessment of Suicide Risk
NGASR-SPN: Nurses’ Global Assessment of Suicide Risk–Spanish version
